# Short and long-term prognosis of admission hyperglycemia in patients with and without diabetes after acute myocardial infarction: a retrospective cohort study

**DOI:** 10.1186/s12933-022-01550-4

**Published:** 2022-06-23

**Authors:** Hanzohra Upur, Jia-Li Li, Xiao-Guang Zou, Yu-Ying Hu, He-Yin Yang, Alimujiang Abudoureyimu, Anwar Abliz, Mamatili Abdukerim, Min Huang

**Affiliations:** 1 Institute of Clinical Pharmacology, School of Pharmaceutical Sciences, Sun Yat-Sen University, Guangzhou, China; 2 Department of Cardiology, The First People’s Hospital of Kashi Prefecture, Kashi, China

**Keywords:** Acute myocardial infarction, Hyperglycemia, Diabetes status, Outcomes

## Abstract

**Objective:**

Admission hyperglycemia is associated with poor prognosis in patients with acute myocardial infarction (AMI), but the effects of baseline diabetes status on this association remain elusive. We aim to investigate the impact of admission hyperglycemia on short and long-term outcomes in diabetic and non-diabetic AMI patients.

**Methods:**

In this retrospective cohort study, 3330 patients with regard to first-time AMI between July 2012 and July 2020 were identified. Participants were divided into two groups according to diabetes status (1060 diabetic patients and 2270 non-diabetic patients). Thereafter, they were divided into four groups according to diabetes status-specific cutoff values of fasting blood glucose (FBG) identified by restricted cubic spline. Short-term outcomes included in-hospital death and cardiac complications. Long-term outcomes were all-cause mortality and major adverse cardiovascular events (MACE). Inverse probability of treatment weighting (IPTW) was conducted to adjust for baseline differences among the groups, followed by a weighted Cox proportional hazards regression analysis to calculate hazard ratios and 95% confidence intervals for all-cause mortality associated with each FBG category. Subgroup analysis and sensitivity analysis were performed to test the robustness of our findings.

**Results:**

During a median follow-up of 3.2 years, 837 patients died. There was a significant interaction between diabetes status and FBG levels for all-cause mortality during long-term follow-up (p-interaction < 0.001). Moreover, restricted cubic spline curves for the association between FBG and all-cause mortality followed a J shape in patients with diabetes and a non-linear in patients without diabetes. Kaplan–Meier analysis demonstrated greater survival in non-hyperglycemia patients compared to hyperglycemia patients for both diabetic and non-diabetic patients groups. Survival of hyperglycemia patients without diabetes greater than in hyperglycemia patients with diabetes. In the weighted Multivariable cox analysis, admission hyperglycemia predicted higher short and long-term mortality. Subgroup analysis and sensitivity analysis showed the robustness of the results.

**Conclusions:**

The inflection points of FBG level for poor prognosis were 5.60 mmol/L for patients without diabetes and 10.60 mmol/L for patients with diabetes. Admission hyperglycemia was identified as an independent predictor of worse short and long-term outcomes in AMI patients, with or without diabetes. These findings should be explored further.

**Supplementary Information:**

The online version contains supplementary material available at 10.1186/s12933-022-01550-4.

## Introduction

Hyperglycemia during hospital admission is common in patients with AMI and independently associated with worse prognosis [1–4], although the association may be nonlinear [5], and data conflict as to whether this association varies by diabetes status [4, 6, 7]. Admission hyperglycemia occurs in 25–50% of patients, depending on the definition of admission hyperglycemia [8]. There is still no consensus on what blood glucose level defines admission hyperglycemia. It is well known that diabetes is a common comorbidity in patients with cardiovascular diseases [9]. Patients with AMI and diabetes show a more than two-fold higher risk for short and long-term mortality than patients without diabetes [10, 11]. Previous studies showed a stronger association between a diagnosis of clinical diabetes and incident mortality in hyperglycemia patients than non-hyperglycemia patients without diabetes when using the same prognostic cutoff value for both diabetic and non-diabetic patients [1]. Contributors to such diabetes status-based differences are not clear, although disparities in the prevalence of uncontrolled blood glucose and mortality are a possibility. Data are also lacking on diabetes status differences in the prognostic relevance of the blood glucose levels for defining admission hyperglycemia.

Although less is known about the association between admission hyperglycemia and mortality by diabetes status, recent studies demonstrated that admission hyperglycemia was an independent predictor of mortality in AMI patients without diabetes when used the same or different cutoff values for diabetic and non-diabetic patients [4, 7]. Data are lacking on diabetes status differences in absolute measures of mortality risk associated with admission hyperglycemia. Therefore, there is a critical need to take patients’ diabetes status into account to avoid incorrect estimation of the real prevalence of admission hyperglycemia.

Studies evaluating diabetes status-based differences in mortality risk associated with admission hyperglycemia have not used the optimal cutoff values of FBG separately for diabetic and non-diabetic patients or demonstrated whether differences in mortality risk persist across varying levels of FBG. Thus, we investigated the severity of hyperglycemia by diabetes status subgroups, differences in the risk of incident mortality across increasing levels of FBG between diabetes status subgroups, and evaluated FBG cutoff values in patients with and without diabetes to establish their value in predicting the short and long-term prognosis of patients with AMI.

## Methods

### Study design and population

This retrospective, population-based cohort study included patients who were admitted to the chest pain center in the First People’s Hospital of Kashi Prefecture for their first AMI between July 2012 and July 2020. AMI was defined according to the current European guidelines [12], as non-ST-elevation myocardial infarction (NSTEMI) or ST-elevation myocardial infarction (STEMI). Exclusion criteria encompassed patients with previous myocardial infarction, missing crucial laboratory data (FBG on admission or glycated hemoglobin [HbA1c]), severe valvular heart diseases, severe renal failure, or tumors. This study was approved by the local institutional review board of First People’s Hospital of Kashi Prefecture (Approval No. 2021-03). All participants provided written informed consent.

### Data collection and definitions

Demographic and clinical data were collected at baseline through electrical medical records review. Demographic data included age, sex, body mass index (BMI), ethnicity. smoking and drinking habits, and family history of cardiovascular disease. Clinical data consisted of diagnosis, medical history, laboratory test results, medications at discharge, and clinical therapies. The FBG levels were assessed within 24 h of admission. Pre-existing diabetes mellitus was defined as a known reported history of diabetes at admission, either treated with diet and lifestyle measures alone or with the additional use of oral glucose-lowering medications and insulin. Newly diagnosed diabetes was defined based on the oral glucose tolerance test, fasting glucose test or glycated hemoglobin ≥ 6.5% during hospitalization. Patients without a history of diabetes and with HbA1c < 6.5% mmol/mol were considered non-diabetic. Pre-existing diabetes mellitus with admission glucose level > 20 mmol/L received insulin–glucose infusion to decrease blood glucose between 7 and 15 mmol/L for the first 24 h, followed by subcutaneous insulin injections. Newly diagnosed diabetes and non-diabetic patients were not received glucose-lowering treatment for the first 24 h. Hyperglycemia was defined as FBG levels ≥ 5.6 mmol/L for non-diabetic patients or ≥ 10.6 mmol/L for diabetic patients.

### Follow-up and outcomes

Patients’ follow-up data, including survival data and clinical event data, were obtained by review of all available medical records, personal communication with the patient’s physician, and a telephone interview with the patient or a patient's close relative conducted by trained personnel. Short-term outcomes included in-hospital death and cardiac complications (ventricular fibrillation, cardiogenic shock, atrial fibrillation, heart failure, and arrhythmia). Long-term outcomes were all-cause mortality and MACE [cardiovascular mortality, re-hospitalization for AMI, target vessel revascularization (TVR), heart failure, and stroke].

### Statistical analyses

Baseline characteristics of the patients by categorical FBG level were summarized using descriptive statistics (frequencies with proportions or means with SDs as appropriate). Demographic and clinical characteristics were compared using the chi-square test for categorical variables and the Kruskal–Wallis test for continuous variables. Missing values at baseline were imputed by using the Multivariate Imputation by Chained Equations (MICE) package in python using random forest imputations. The median follow-up time was estimated by using the reverse Kaplan Meier method.

Cox proportional hazard models were used to assess the association between FBG and mortality, both with FBG as a categorical variable (to account for the glycemic threshold) and then as a restricted cubic spline, to explore a potential nonlinear relationship between FBG and mortality.

#### FBG as a restricted cubic spline

For FBG as a restricted cubic spline, we performed Cox proportional hazards regression with mortality as the outcome. The relative hazards of mortality (with FBG of 5.6 mmol/L for non-diabetic and 10.6 mmol/L for diabetic patients as reference) were graphed, stratified by diabetes status. Splines were modeled by restricted cubic splines with 3 knots at the 10th, 50th, and 90th percentiles.

#### FBG as a categorical variable

For FBG as a categorical variable, the 4 categories of FBG were chosen a priori based on the optimal cutoff values determined by our restricted cubic spline analysis: non-hyperglycemia patients without diabetes (< 5.6 mmol/L), hyperglycemia patients without diabetes (≥ 5.6 mmol/), non-hyperglycemia patients with diabetes (< 10.6 mmol/L), and hyperglycemia patients with diabetes (≥ 10.6 mmol/L). Kaplan–Meier survival curves were used for survival analysis. We applied IPTW to the Cox models for all-cause mortality to adjust for baseline differences. The treatment probabilities were calculated from a logistic regression using a set of covariates deemed to have affected baseline differences, including patient characteristics, medical history, baseline assessments Systolic, laboratory values, revascularization, and medications. Hazard ratios (HRs) or odds ratios (ORs) with 95% confidence intervals (CIs) for the outcomes according to FBG categories were calculated using Cox proportional hazards models, all of which were inverse probability of treatment weighted. Model 1 included FBG categories only. Model 2 included FBG categories, age (≤ 55 and > 55 years), gender, ethnic, hypertension, diagnosis (NSTEMI and STEMI), Killip class (< II and ≥ II), and EF (< 40 and ≥ 40). Model 3 included FBG categories, age, gender, Killip class, ethnic, drinking, hypertension, COPD, liver disease, lung disease, diagnosis, EF, PCI, CABG, ACE inhibitor/ARB, and beta-blocker. These variables were the multiple Cox proportional hazard model, derived from the LASSO method and stepwise backward selection procedures. We performed tests for linear trends by entering the median value of each category of FBG level as a continuous variable in the models.

In addition, to examine the presence or absence of covariates differences in outcomes, we conducted subgroup analyses by age (≤ 55 and > 55 years), gender (male and female), ethnic (Uyghur and Han), hypertension (no and yes), diagnosis (NSTEMI and STEMI), Killip class (< II and ≥ II), and EF (< 40 and ≥ 40). Tests of interaction were used to assess the differences in FBG levels across these subgroups. To assess the robustness of the study findings, we conducted a series of sensitivity analyses. First, nonlinear associations between FBG and all-cause mortality were further examined using restricted cubic splines with multivariable-adjusted Cox proportional hazards models. Second, the first sensitivity analysis was repeated with the time to MACE instead of time to mortality. Third, we performed the restricted cubic spline analysis after excluding the participants who died during hospitalization (n = 221). Fourth, we conducted Cox proportional hazard models without applying IPTW. Models with significant interaction terms were visualized using the visreg package. All reported p-values were 2-tailed, and a p-value ≤ 0.05 was considered statistically significant. All statistical analyses were performed using IBM SPSS statistics (version 25), R (version 4.1.2), and Python (version 3.10.0).

### Results

#### FBG level categorization

**Fig. 1:**
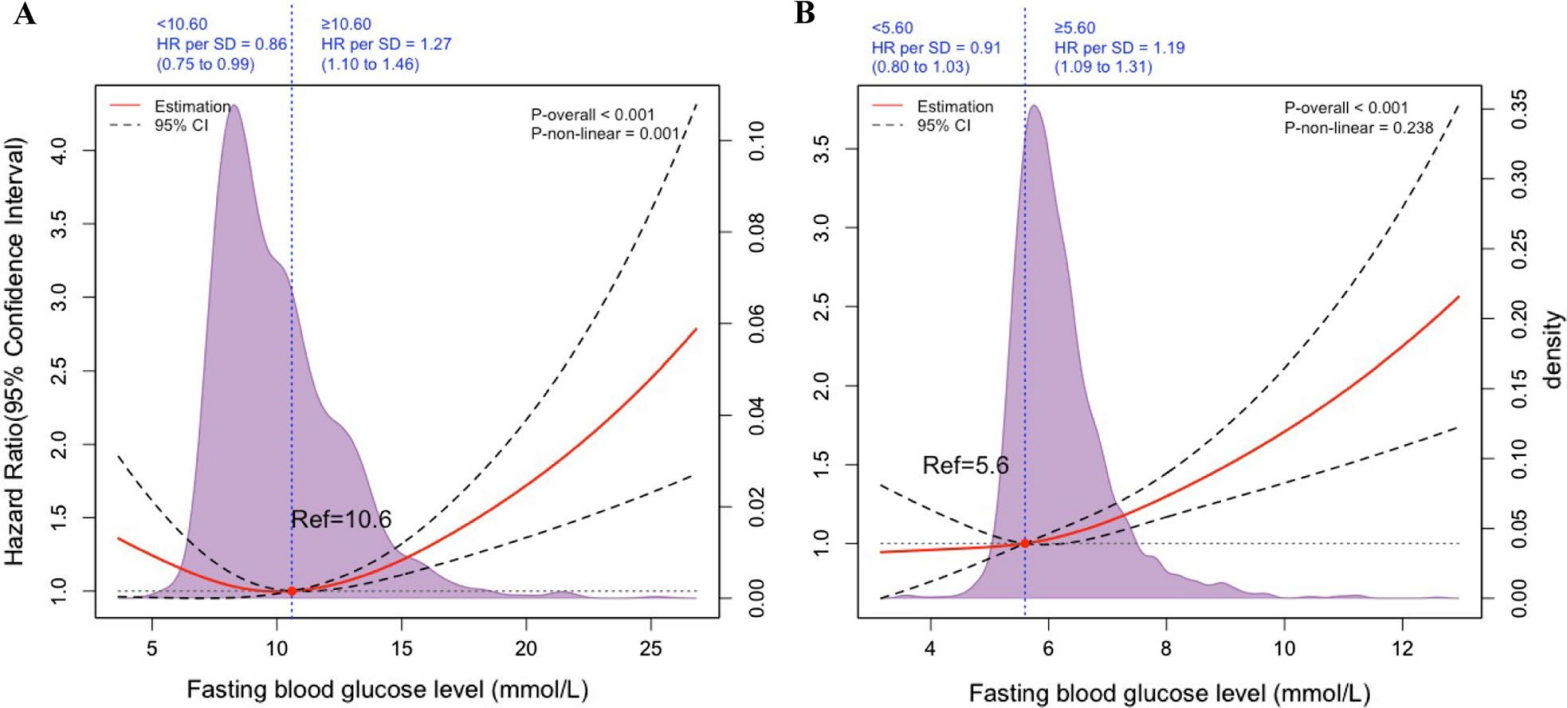
Hazard Ratios of All-Cause Mortality According to FBG levels in AMI patients. **A** non-diabetic patients and all-cause mortality. **B** diabetic patients and all-cause mortality. Solid red lines are hazard ratios and dashed lines show 95% confidence intervals based on restricted cubic spline regressions. Reference line for no association (hazard ratio: 1.0) is indicated by dashed grey line while areas of purple show fraction of population at different FBG concentrations. The red points for hazard ratio = 1

There was a significant interaction between diabetes status and increasing FBG levels on all-cause mortality (p < 0.001), with higher FBG levels being associated with greater all-cause mortality among non-diabetic than among diabetic patients (Additional file 1: Figure S2). Therefore, these analyses were stratified according to diabetes status and defined diabetes status-specific FBG cutoff values. Restricted cubic spline analysis was performed to evaluate cutoff values for FBG levels associated with all-cause mortality in patients with and without diabetes (Fig. 1). The risk of all-cause mortality was relatively flat and the hazard ratio was < 1 until around 5.60 mmol/L (100 mg/dL) of predicted FBG level and then started to increase rapidly afterwards (p for overall < 0.001 and p for non-linearity < 0.238) in patients without diabetes. Above 5.6 mmol/L, the hazard ratio per standard deviation higher predicted all-cause mortality was 1.19 (1.09–1.31). We observed a J shaped association between FBG and all-cause mortality in patients with diabetes, the plot showed a substantial reduction of the risk within the lower range of predicted FBG level, which reached the lowest risk around 10.6 mmol/L (190 mg/dL) and then increased thereafter (p for overall < 0.001 and p for non-linearity < 0.001). Above 10.6 mmol/L, the hazard ratio per standard deviation higher predicted all-cause mortality was 1.27 (1.10–1.46). Accordingly, we chose FBG of 5.60 mmol/L for patients without diabetes and 10.60 mmol/L for patients with diabetes as convenient cutoff values to classify patients into four groups.

### Baseline characteristics

A total of 3330 patients with first-time AMI were enrolled in our study. The mean age of participants was 56.3 (SD 12.3) years, 78% of participants were male, 87% of participants were Uyghur, and 32% of participants were diabetes. Among the patients, 1091 (33%) were classified as non-hyperglycemia without diabetes, 1179 (35%) as hyperglycemia without diabetes, 635 (19%) as non-hyperglycemia with diabetes, and 425 (13%) as hyperglycemia with diabetes (Additional file 1: Fig. S1).

The patient characteristics were compared between groups categorized by FBG level (Table 1). Among both diabetic and non-diabetic patients, hyperglycemia patients tended to have higher heart rate, Killip class, procalcitonin, c-reactive protein, triglyceride, HDL cholesterol, LDL cholesterol, apolipoprotein A, and peak hs Troponin I. Among the non-diabetic patients, total cholesterol was lower in hyperglycemia patients, while hyperglycemia patients had higher in diabetic patients. In the non-diabetic patients, higher uric acid and a greater prevalence of STEMI were more often observed in hyperglycemia patients, while no statistically significant differences were detected in the diabetic patients. When comparing the two hyperglycemia subgroups, hyperglycemia patients without diabetes tended to have more frequent of younger, men, former smokers, and STEMI; were more likely to have less frequent of hyperglycemia, liver disease, and lung disease; exhibited lower Killip class, heart rate, procalcitonin, c-reactive protein, and triglyceride; had greater Left ventricular ejection fraction value, haemoglobin, glomerular filtration rate, HDL cholesterol, apolipoprotein A, uric acid, and peak hs Troponin I.

**Table 1:** Baseline characteristics

	Patients without diabetes			Patients with T2DM			Hyperglycemia diabetes vs non-diabetes
	Non-hyperglycemia (n = 1091)	Hyperglycemia (n = 1179)	p-value	Non-hyperglycemia (n = 635)	Hyperglycemia (n = 425)	p-value	p-value
Patient characteristics							
Age, years			0.736			0.485	0.000
≤ 55	590 (54)	629 (53)		258 (41)	182 (43)		
> 55	501 (46)	550 (47)		377 (59)	243 (57)		
Male sex	903 (83)	965 (82)	0.582	456 (72)	279 (66)	0.035	< 0.0001
BMI, Kg/m^2^	25.95 [23.53, 28.23]	26.06 [23.73, 27.98]	0.713	26.16 [24.22, 28.52]	26.06 [23.57, 28.65]	0.264	0.547
Ethnic			0.424			0.611	0.167
Uyghur	946 (87)	1015 (86)		555 (87)	373 (88)		
Han	120 (11)	144 (12)		73 (11)	50 (12)		
Others	25 (2)	20 (2)		7 (1)	2 (0)		
Smoking status			0.498			0.091	< 0.0001
Never smoker	545 (50)	607 (51)		380 (60)	282 (66)		
Former smoker	470 (43)	481 (41)		203 (32)	111 (26)		
Current smoker	76 (7)	91 (8)		52 (8)	32 (8)		
Drinking	144 (13)	156 (13)	1.000	61 (10)	41 (10)	1.000	0.058
Killip class			< 0.0001			0.002	< 0.0001
< II	543 (50)	448 (38)		223 (35)	110 (26)		
≥ II	548 (50)	731 (62)		412 (65)	315 (74)		
LVEF, %			0.193			0.537	0.003
< 40	92 (8)	119 (10)		90 (14)	66 (16)		
≥ 40	999 (92)	1060 (90)		545 (86)	359 (84)		
STEMI	730 (67)	885 (75)	< 0.0001	399 (63)	277 (65)	0.473	0.000
Medical history							
Hypertension	393 (36)	439 (37)	0.571	328 (52)	220 (52)	1.000	< 0.0001
Stroke	149 (14)	143 (12)	0.287	91 (14)	61 (14)	1.000	0.236
Prior CAD	20 (2)	32 (3)	0.206	22 (3)	12 (3)	0.599	0.864
COPD	50 (5)	42 (4)	0.242	34 (5)	19 (4)	0.567	0.459
Liver disease	15 (1)	21 (2)	0.503	32 (5)	26 (6)	0.492	< 0.0001
Lung disease	7 (1)	7 (1)	1.000	7 (1)	9 (2)	0.205	0.018
Baseline assessments systolic							
Systolic BP, mmHg	123 [110, 140]	124 [110, 140]	0.653	125 [112, 140]	129 [110, 140]	0.829	0.193
Diastolic BP, mmHg	79 [70, 89]	79 [70, 90]	0.586	80 [70, 90]	80 [70, 90]	0.713	0.642
Heart rate, bpm	80 [70, 88]	85 [74, 98]	< 0.0001	84 [75, 95]	92 [80, 103]	< 0.0001	< 0.0001
Laboratory values							
Haemoglobin, g/L	145 [134, 156]	149 [137, 159]	0.000	142 [128, 155]	143 [129, 155]	0.628	< 0.0001
Procalcitonin, ng/mL	0.05 [0.03, 0.09]	0.06 [0.04, 0.12]	< 0.0001	0.06 [0.04, 0.13]	0.07 [0.04, 0.20]	0.001	< 0.0001
C-reactive protein, mg/L	5.48 [1.51, 19.87]	11.35 [3.30, 35.29]	< 0.0001	9.94 [2.21, 31.01]	17.84 [4.79, 52.54]	< 0.0001	0.000
eGFR, mL/min/1.73 m^2^	104.37 [82.04, 129.46]	102.92 [78.87, 130.34]	0.175	94.46 [72.67, 123.28]	93.39 [63.49, 119.96]	0.118	< 0.0001
Total cholesterol, mmol/L	1.33 [1.01, 1.86]	1.25 [0.92, 1.77]	< 0.0001	1.40 [1.07, 2.01]	1.61 [1.16, 2.27]	< 0.0001	0.800
Triglyceride, mmol/L	3.81 [3.24, 4.45]	4.16 [3.48, 4.76]	0.000	3.83 [3.16, 4.54]	4.16 [3.44, 4.92]	0.001	< 0.0001
HDL cholesterol, mmol/L	2.46 [2.00, 3.01]	2.76 [2.22, 3.32]	< 0.0001	2.47 [1.92, 3.10]	2.67 [2.16, 3.34]	0.037	< 0.0001
LDL cholesterol, mmol/L	0.88 [0.75, 1.03]	0.97 [0.82, 1.15]	< 0.0001	0.86 [0.70, 1.03]	0.89 [0.73, 1.07]	0.000	0.357
Apolipoprotein A, g/L	1.00 [0.88, 1.15]	1.04 [0.91, 1.18]	0.000	0.98 [0.84, 1.13]	1.01 [0.86, 1.18]	0.018	0.021
Uric acid, μmol/L	311 [253, 384]	323 [264, 395]	0.002	319 [255.600, 397.500]	304 [232, 399]	0.061	0.004
Peak hs Troponin I, ng/mL	4.75 [0.51, 21.73]	14.75 [2.66, 42.24]	< 0.0001	5.09 [0.92, 20.43]	10.45 [1.56, 34.57]	< 0.0001	0.007
Revascularization							
PCI	584 (54)	683 (58)	0.038	325 (51)	210 (49)	0.574	0.003
CABG	4 (0)	2 (0)	0.436	1 (0)	1 (0)	1.00	1.000
Medications							
Statin	1076 (99)	1148 (97)	0.037	625 (98)	414 (97)	0.266	1.000
Platelet inhibitor	1074 (98)	1159 (98)	0.869	626 (99)	420 (99)	0.792	0.648
ACE inhibitor/ARB	771 (71)	783 (66)	0.030	470 (74)	294 (69)	0.094	0.307
Beta-blocker	883 (81)	916 (78)	0.062	510 (80)	337 (79)	0.696	0.538

Continuous variables are presented as median (IQR) while categorical ones as n (%)

*BMI* body max index, *LVEF* left ventricular ejection fraction, *STEMI* ST-segment elevation myocardial infarction, *CAD* coronary artery disease, *COPD* chronic obstructive pulmonary disease, *SBP* systolic blood pressure, *DBP* diastolic blood pressure, *eGFR* estimated glomerular filtration rate, *LAD* left anterior descending coronary, *LCX* left circumflex artery, *RCA* right coronary artery, *PCI* percutaneous coronary intervention, *CABG* coronary artery bypass grafting, *ACEI* angiotensin-converting enzyme inhibitor, *ARB* angiotensin-receptor blocker

### FBG and clinical outcomes

**Fig. 2:**
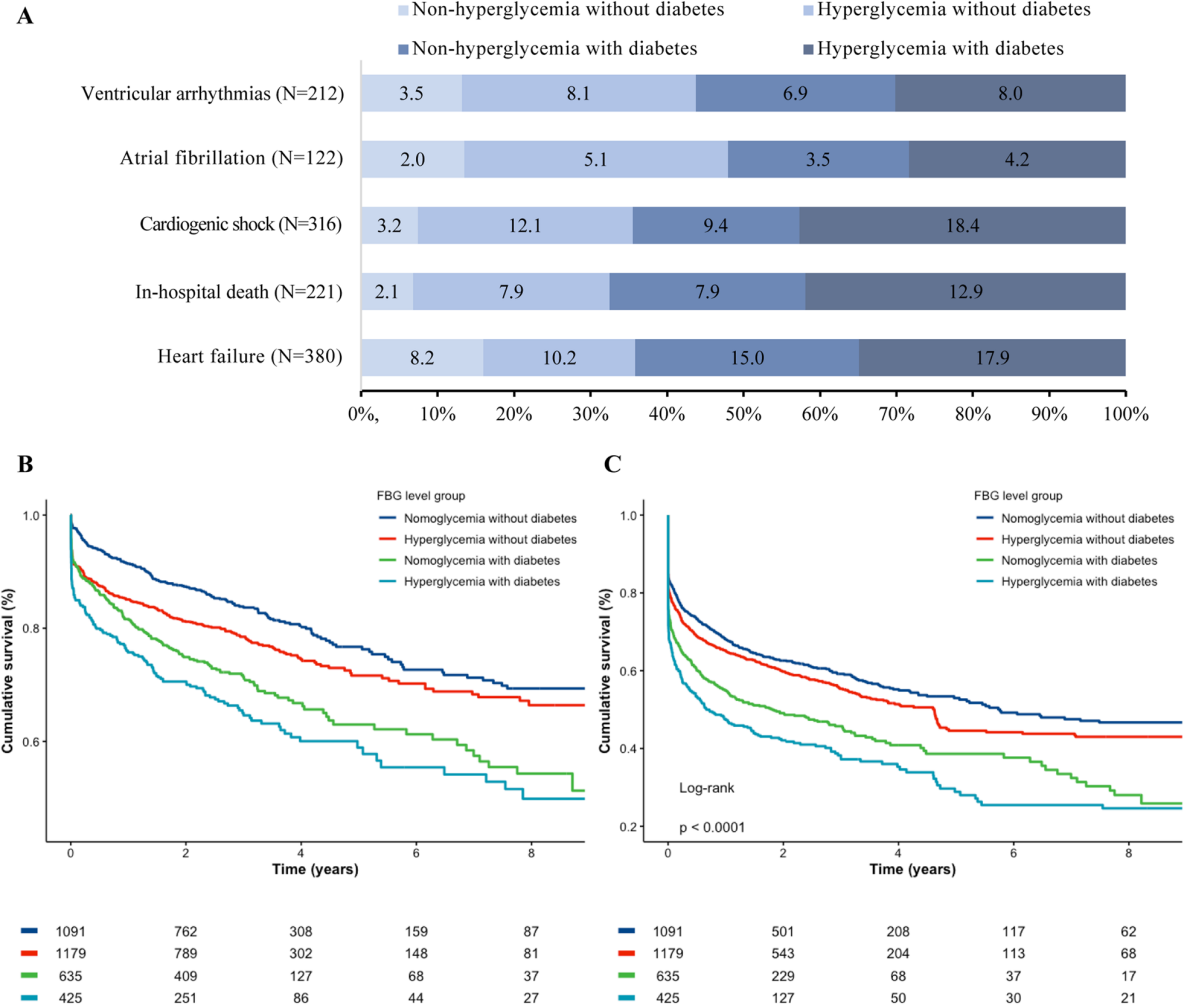
Short and long-term outcomes of AMI patients, according to FBG levels. **A** Stacked bar chart of short-term outcomes. **B** Kaplan–Meier survival curves of all-cause mortality. **C** Kaplan–Meier survival curves of major adverse cardiovascular event (MACE)

During hospitalization, 221 deaths occurred. Among both diabetic and non-diabetic patients, in-hospital death and cardiogenic shock occurred more frequently among hyperglycemia patients (p < 0.01). In the non-diabetic patients, hyperglycemia patients exhibited a greater arrhythmogenic burden (atrial fibrillation and ventricular arrhythmias) during hospitalization when compared to non-hyperglycemia patients (p < 0.01). In-hospital death, heart failure, and cardiogenic shock occurred more frequently among hyperglycemia patients with diabetes compared to hyperglycemia patients without diabetes (p < 0.01, Fig. 2).

During a median follow-up period of 3.2 years, 837 deaths (756 from cardiovascular causes) and 1600 MACE were recorded. Kaplan–Meier survival analysis showed a significant difference in the incidence of all-cause mortality and MACE among the four groups(p < 0.001). In non-diabetic patients, all-cause mortality occurred more frequently among hyperglycemia patients (p = 0.003). In both non-diabetic and diabetic patients, MACE occurred more frequently among hyperglycemia patients (p = 0.053 and p = 0.018). When comparing the two hyperglycemic subgroups, all-cause mortality and MACE were more often observed in hyperglycemia patients with diabetes (p < 0.001, Fig. 2).

**Fig. 3:**
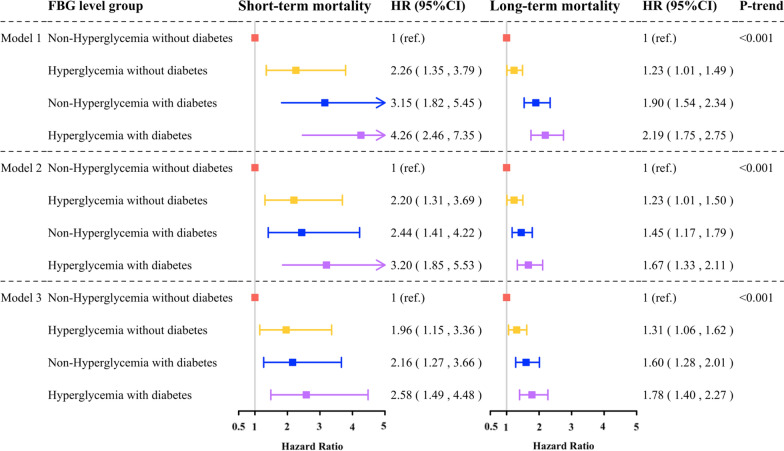
Risk for short and long-term mortality according to FBG levels. All models were inverse probability of treatment weighted (IPTW). IPTW included all the clinical variables listed in Table 1. Model 1 included FBG categories only. Model 2 included FBG categories, age, gender, ethnic, hypertension, diagnosis, Killip class, and EF. Model 3 included FBG categories, age, gender, Killip class, ethnic, drinking, hypertension, COPD, liver disease, lung disease, diagnosis, EF, PCI, CABG, ACE inhibitor/ARB, and beta-blocker. *CI* confidence interval; *HR* hazard ratio. Test for trend based on variable containing median value for each quintile

IPTW Cox models were performed to calculate the HRs of mortality across FBG categories. In a weighted univariable Cox model, hyperglycemia (non-diabetic patients: HR 2.26, 95% CI 1.35–3.79; diabetic patients: HR 1.70, 95% CI 1.16–2.49) was a significant predictor of short-term mortality (in-hospital death). As for the long-term outcome, hyperglycemia (non-diabetic patients: 1.23; 95% CI 1.01–1.49; diabetic patients: HR 1.23, 95% CI 1.00–1.58) was identified as an independent predictor of long-term mortality (all-cause mortality). Additional adjustment for age, gender, ethnic, hypertension, diagnosis, Killip class, and EF (model 2) or the clinical variables from the multiple Cox proportional hazard model (c-indices of the model was 0.81) (model 3) did not alter the significance of the results. Moreover, there was a consistent dose–response relationship between the FBG and short and long-term mortality (p for trend < 0.001 for all) (Fig. 3).

### Subgroup and sensitivity analysis

Subgroup analyses of risk for all-cause mortality based on demographics and clinical characteristics and FBG categories showed consistent results across different subgroups (p for interaction > 0.05 for all, Additional file 1: Fig. S3).

Multivariable-adjusted restricted cubic splines showed similar trends for continuous levels of FBG and risk of all-cause mortality and MACE among patients with and without diabetes. Reimplementing Multivariable-adjusted restricted cubic spline analysis after excluding participants who died during hospitalization resulted in a similar shaped association between FBG and all-cause mortality among patients with and without diabetes (Additional file 1: Fig. S4). Multivariable analysis without IPTW also yielded consistent results with those of the main analyses (Additional file 1: Fig. S5).

### Discussion

We investigated the diabetes status-specific risks of mortality across increasing levels of FBG, a key glycemic maker in identifying a group of “high-risk” patients who could probably benefit from a proper secondary prevention medical therapy. When treating FBG as a restricted cubic spline term, our findings demonstrated that the association between FBG and all-cause mortality followed a J shape in patients with diabetes and a non-linear in patients without diabetes. The HRs of mortality significantly increased when FBG levels ≥ 5.60 mmol/L for patients without diabetes and ≥ 10.60 mmol/L for patients with diabetes. Based on FBG categories, our findings demonstrated that admission hyperglycemia was independently associated with short and long-term outcomes in AMI patients, regardless of diabetes status.

In this study, we observed that elevated fasting blood glucose in AMI has a strong association with worse outcomes. Previous studies have primarily focused on the prognosis value of admission hyperglycemia in both patients with and without diabetes [1–4]. Some studies showed that persisting hyperglycaemia was a more accurate and stronger independent predictor of risk than admission blood glucose. Hyperglycaemia (blood glucose ≥ 8.9 mmol/L), persists from admission to at least 24 h after symptom onset, is associated both with reduced myocardial perfusion despite patency of the infarct-related artery and with pre-discharge left ventricular impairment [13]. In another study, persistent hyperglycemia in myocardial infarction has a stronger relation with 30-day MACE than elevated glucose at admission [14]. Fasting glucose was superior to admission glucose with regard to 30-day mortality in previous study [15]. The superiority of FBG over random glucose levels in predicting outcome probably results from factors such as differences in the amount of caloric intake and time since the last meal. In a recent study, random blood glucose and FBG were positively correlated with the Gensini score in AMI patients, and FBG was an independent risk factor for the Gensini score in AMI patients [16]. These findings demonstrate that acute and continuing elevation of blood glucose, rather than an underlying “diabetic state”, may promote microvascular dysfunction, contributing to poorer outcomes. In 1219 non-diabetic patients after AMI, higher admission glucose concentrations predicted greater mortality, while larger reductions in glucose over 24 h predicted lower 30-day mortality. Baseline glucose and the 24-h change in glucose remained significant predictors of 180-day death [17]. Therefore, we considered that FBG levels at admission may better represent the continuing elevation of blood glucose and a greater potential predictor of clinical outcomes in AMI patients.

There are no accurate cutoff values of blood glucose in AMI patients to predict adverse events or accurate definition for admission hyperglycemia with AMI. Prior studies elevated that Stress Hyperglycaemia Ratio (SHR), hemoglobin a1c (HbA1c), and glucose-HbA1c-ratio (GHR) were reported as predictors of clinical outcomes in AMI and other pathological processes. Still, their optimal values were not clarified as thresholds have been arbitrarily selected [4, 18, 19]. In this study, we found a significant interaction between diabetes status and increasing FBG levels on all-cause mortality. This finding is similar to that of a previous study and found a significant interaction between diabetes status and increasing blood glucose levels on mortality. We also identified the nonlinear association between the FBG and all-cause mortality in patients with or without diabetes. The optimal cutoff values for FBG levels were 10.6 mmol/L for diabetic patients and 5.6 mmol/L for non-diabetic patients, above which there was a statistically significant increase in mortality. Our study corroborates these findings that there are different cutoff values for blood glucose to predict outcomes depending on the diabetes status [2, 7].

This study demonstrated that patients with higher FBG levels (≥ 10.6 mmol/L for diabetic patients and ≥ 5.6 mmol/L for non-diabetic patients) had higher heart rate, Killip class, procalcitonin, c-reactive protein, triglyceride, HDL cholesterol, LDL cholesterol, apolipoprotein A, and a greater prevalence of in-hospital death and cardiogenic shock than those with lower FBG levels. The finding indices are paralleled by a higher GRACE score observed in hyperglycemic subjects, both diabetic and non-diabetic groups. Our findings are consistent with those of previous studies, which whether patients are diagnosed with diabetes or not, elevated blood glucose concentrations at the time of hospital admission are independently associated with a higher risk of in-hospital mortality and in-hospital complications, such as cardiogenic shock, and arrhythmia in hyperglycemia patients compared with non-hyperglycemia patients without diabetes [1, 20–22]. Several explanations have been proposed for the association between hyperglycemia and adverse events, including hyperglycemic status, or diabetes, affects the pro-inflammatory/oxidative properties and the pro-thrombotic properties in the arterial plaque lesion [23, 24].

In terms of long-term prognosis, hyperglycemia patients in both diabetic and non-diabetic groups exhibited a higher rate of all-cause and MACE. Similarly, hyperglycemia patients with diabetes had significantly higher rates of heart failure [25] and macrovascular complications (stroke and re-AMI). These results might confirm recent studies which demonstrated that Over-inflammation, chronic or acute hyperglycemia, insulin resistance, hyperinsulinemia, and dyslipidemia in T2DM patients, together with decreased levels of high density lipoprotein (HDL) and increased levels of low density lipoprotein (LDL), represent the triggering causes of endothelial disfunction (i.e., impaired balance between the vasoconstriction and vasodilatory properties of endothelium) [26, 27]. The management of hyperglycemic patients during AMI is unclear. An earlier study demonstrated a positive effect of tight glycaemic control, associated to the achievement of glucose target, compared to standard therapy on myocardial injury in hyperglycaemic patients addressed to pPCI [28]. A recent study reported that thrombus aspiration during the primary percutaneous intervention (pPCI) for STEMI reduced clinical outcomes in hyperglycaemic patients, whereas it did not in normoglycaemic ones [24].

We found that hyperglycemia was an independent predictor of all-cause mortality during long-term follow-up which corroborates the key finding from a recent report based on the optimal values (140 mg/dL) for glucose in patients with and without diabetes [4]. Similar findings have also been reported admission hyperglycemia is an independent predictor of in-hospital mortality (admission hyperglycemia defined as ≥ 200 mg/dL) and long-term prognosis (the optimal FBG cutoff values were 14.80 and 6.77 mmol/L for patients with and without diabetes, respectively) in non-diabetic patients after AMI. However, those studies had conflicting results in patients with diabetes, no difference was observed between the hyperglycemic and non-hyperglycemic groups, which might be due to the small sample size or low cutoff value [4, 7].

### Study strengths and limitations

A major strength of our study is the report of both relative estimates of the association between elevated FBG and mortality in diabetic status subgroups as well as absolute mortality risks according to diabetes status and specific FBG levels. To our knowledge, this is the first study verifying the J-shaped or nonlinear association between the FBG and clinical outcomes in AMI patients with and without diabetes. Participants were classified into 4 groups based on the optimal cutoff values of FBG. In addition, we were able to adjust for a wide range of baseline covariates using the IPTW method. Last, short and long-term outcomes were analyzed simultaneously, all of which yielded consistent results. Subgroup analysis, as well as sensitivity analysis, further supported the robustness of our findings. Our study has several limitations. First, due to the long enrollment period, guidelines and recommendations on diagnosis and treatment of AMI have changed over the years which might also influence results of the outcomes. Second, the FBG levels were measured after the PCI procedure in a very small subset of AMI patients. Third, admission hyperglycemia levels may have been influenced by multiple factors such as last meal composition and timing and day versus night measurements.

### Conclusions

According to diabetes status and specific fasting blood glucose levels, our findings demonstrated admission hyperglycemia is an important predictor of short and long-term outcomes in AMI patients, regardless of diabetes status. Subgroup-specific measurements of the relative increase in mortality risk are critical to understanding potential diabetes status differences in the relative contribution of disordered glucose metabolism to mortality risk after AMI. Further research is need to identify how such differences might be incorporated into clinical guidelines the use of diabetes status-specific treatment recommendations across the spectrum of FBG.

## Supplementary Information

**Additional file 1: Figure S1.** Flow chart of the study. **Figure S2.** Significant interactions between diabetes status and FBG levels for all-cause mortality. **Figure S3.** Subgroup Analyses of the Risk for All-Cause Mortality. **Figure S4.** Association between FBG and outcomes using restricted cubic splines with multivariable-adjusted Cox proportional hazards models. **Figure S5.** Risk for short and long-term mortality according to FBG levels.

## Data Availability

The datasets analysed during the current study are available from the corresponding author on reasonable request.

## Abbreviations

AMI: Acute myocardial infarction
FBG: Fasting blood glucose
BMI: Body mass index
CI: Confidence interval
HR: Hazard ratio
IPTW: Inverse probability of treatment weighted
LVEF: Left ventricular ejection fraction
STEMI: ST-segment elevation myocardial infarction
CVD: Cardiovascular disease
COPD: Chronic obstructive pulmonary disease
SBP: Systolic blood pressure
DBP: Diastolic blood pressure
eGFR: Estimated glomerular filtration rate
LAD: Left anterior descending coronary
LCX: Left circumflex artery
RCA: Right coronary artery
PCI: Percutaneous coronary intervention
CABG: Coronary artery bypass grafting
ACEI: Angiotensin-converting enzyme inhibitor
ARB: Angiotensin-receptor blocker
