# Supplementary material for: Short and long-term prognosis of admission hyperglycemia in patients with and without diabetes after acute myocardial infarction: a retrospective cohort study
Source: Cardiovasc Diabetol. 2022 Jun 23;21:114. doi: 10.1186/s12933-022-01550-4 (PMC9229884; doi:10.1186/s12933-022-01550-4)
Supplement: Supplementary file 1 — Additional file 1: Figure S1. Flow chart of the study. Figure S2. Significant interactions between diabetes status and FBG levels for all-cause mortality. Figure S3. Subgroup Analyses of the Risk for All-Cause Mortality. Figure S4. Association between FBG and outcomes using restricted cubic splines with multivariable-adjusted Cox proportional hazards models. Figure S5. Risk for short and long-term mortality according to FBG levels. [file 12933_2022_1550_MOESM1_ESM.pdf]

Online-Only Additional file

Short and long-term prognosis of admission hyperglycemia in patients with and without diabetes after acute myocardial infarction: a retrospective cohort study

Supplementary Figure S1 Flow chart of the study

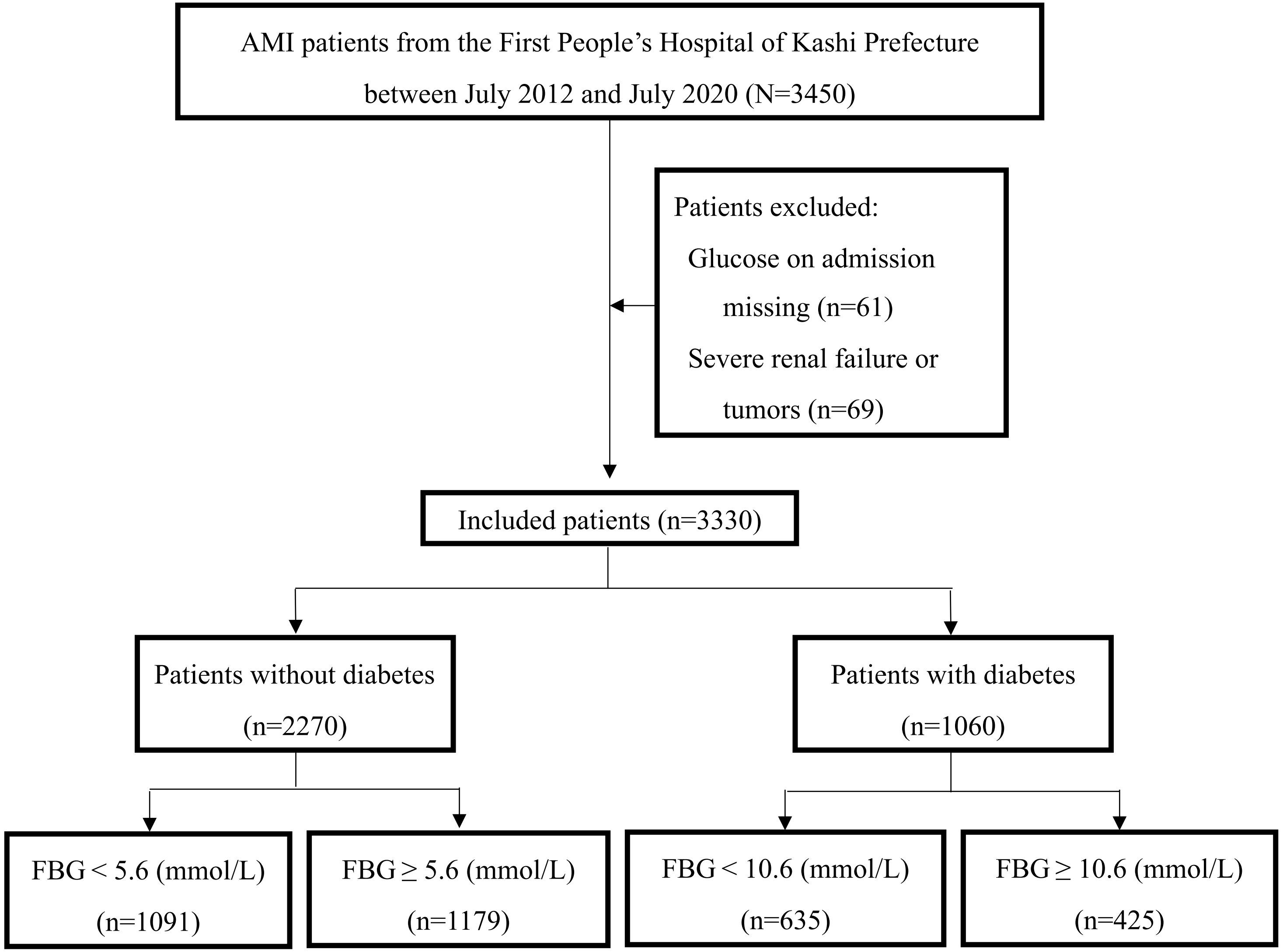

Supplementary Figure S2 Significant interactions between diabetes status and FBG levels for all-cause mortality

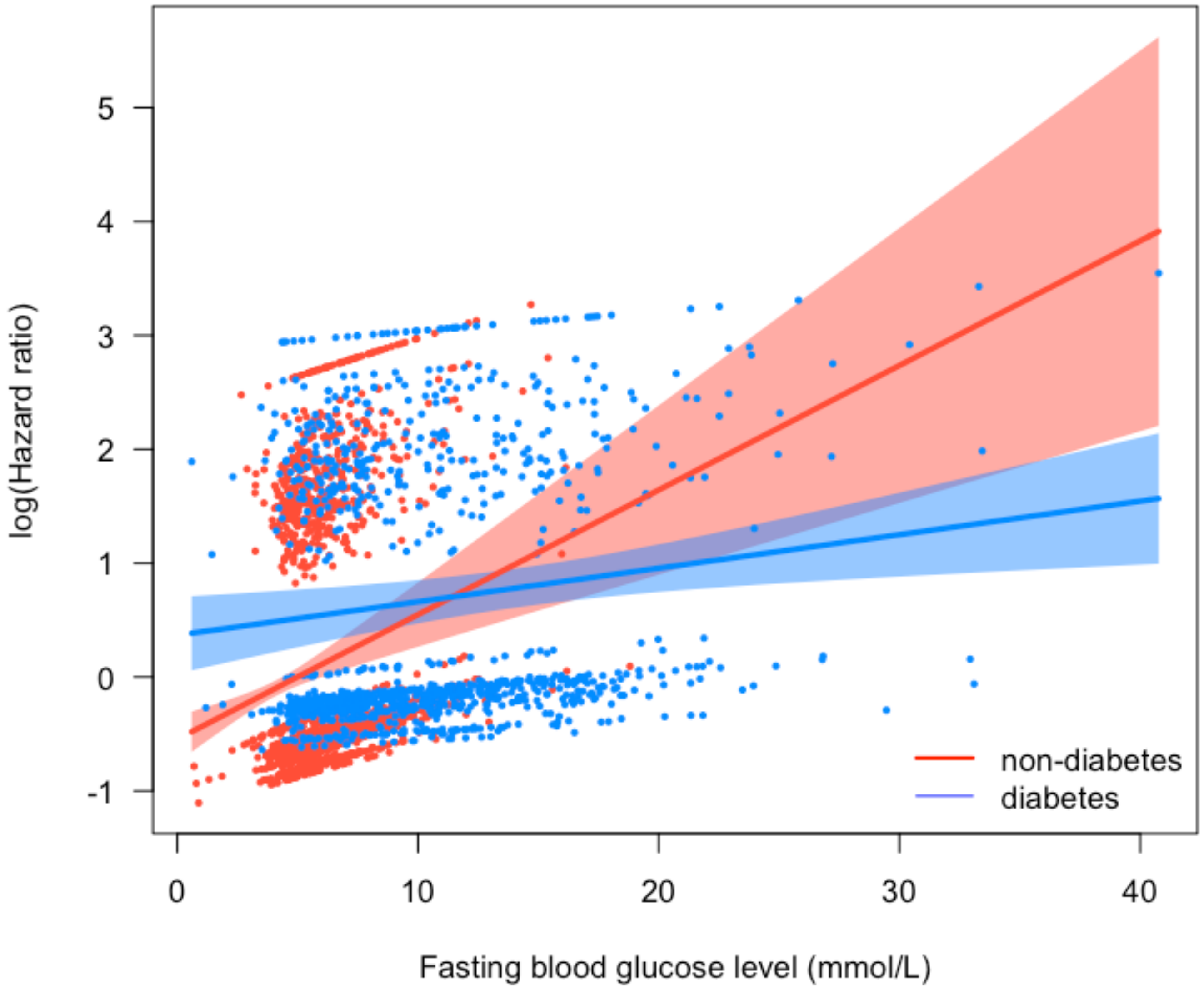

Solid red lines are hazard ratios and **Red shadow** show 95% confidence intervals in non-diabetes patients. **Solid blue lines** are hazard ratios and **Blue shadow** show 95% confidence intervals in diabetes patients.

Supplementary Figure S3 Subgroup Analyses of the Risk for All-Cause Mortality

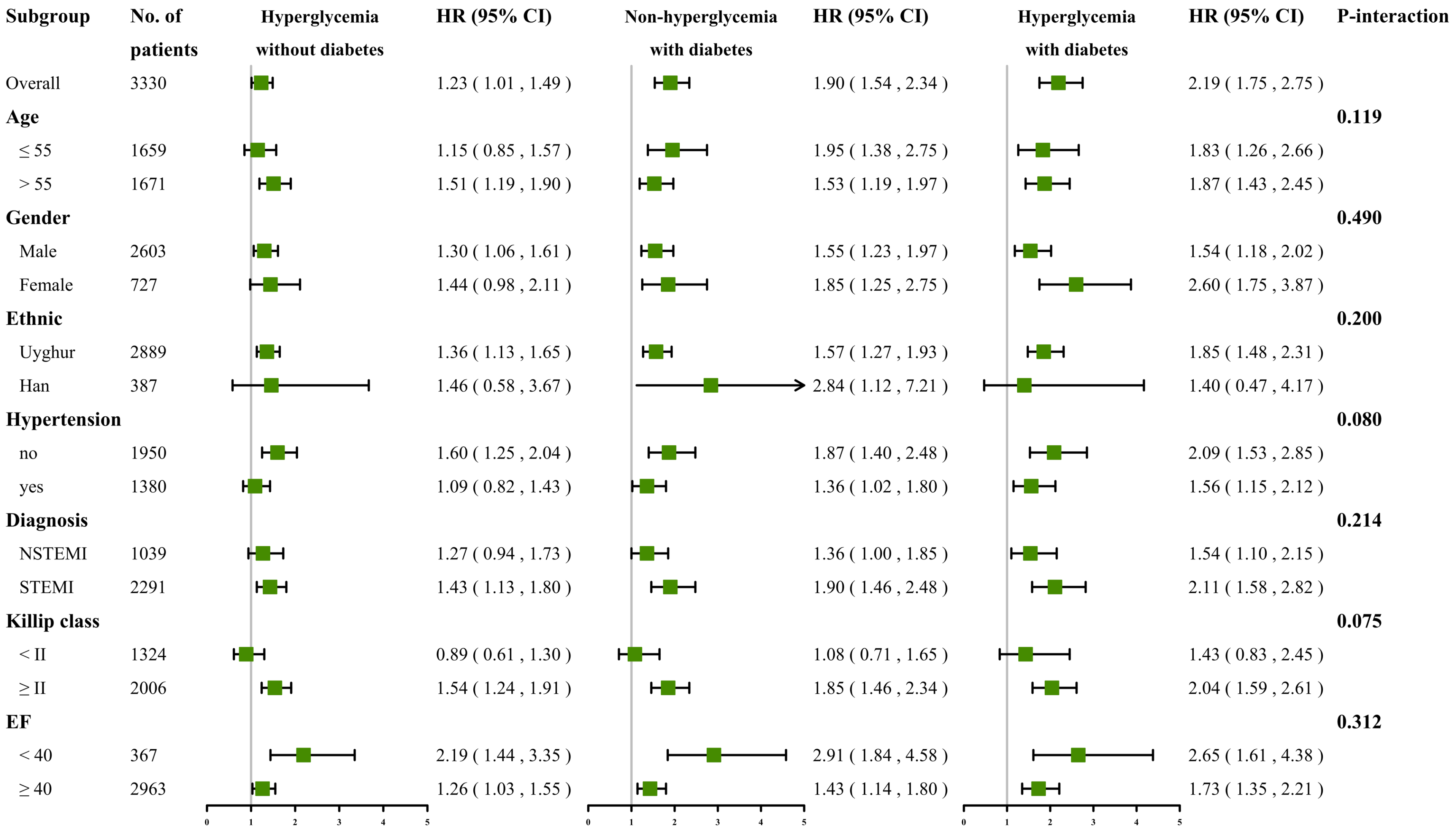

This model is adjusted for FBG categories, age (≤ 55 and > 55 years), gender, Killip class (< II and ≥ II), ethnic, drinking, hypertension, COPD, liver disease, lung disease, diagnosis (NSTEMI and STEMI), EF (< 40 and ≥ 40), PCI, CABG, ACE inhibitor/ARB, and beta-blocker. CI = confidence interval; HR = hazard ratio.

Supplementary Figure S4 Association between FBG and outcomes using restricted cubic splines with multivariable-adjusted Cox proportional hazards models

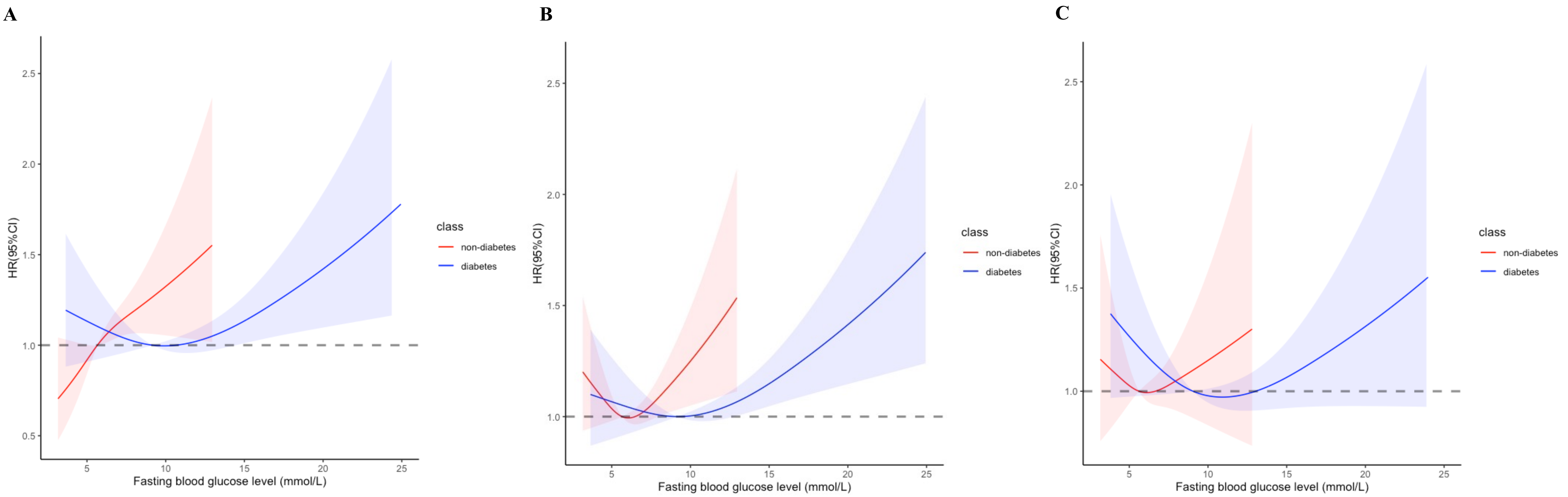

A: FBG and all-cause mortality. **B**: FBG and MACE**. C**: FBG and all-cause mortality (excluding the participants who died during hospitalization). **Solid red lines** are hazard ratios and **Red shadow** show 95% confidence intervals based on restricted cubic spline regressions in non-T2DM patients. **Solid blue lines** are hazard ratios and **Blue shadow** show 95% confidence intervals based on restricted cubic spline regressions in T2DM patients. Reference line for no association (hazard ratio: 1.0) is indicated by **dashed grey line**. All models adjusting for age (≤ 55 and > 55 years), gender, Killip class (< II and ≥ II), ethnic, drinking, hypertension, COPD, liver disease, lung disease, diagnosis (NSTEMI and STEMI), EF (< 40 and ≥ 40), PCI, CABG, ACE inhibitor/ARB, and beta-blocker.

Supplementary Figure S5 Risk for short and long-term mortality according to admission FBG levels

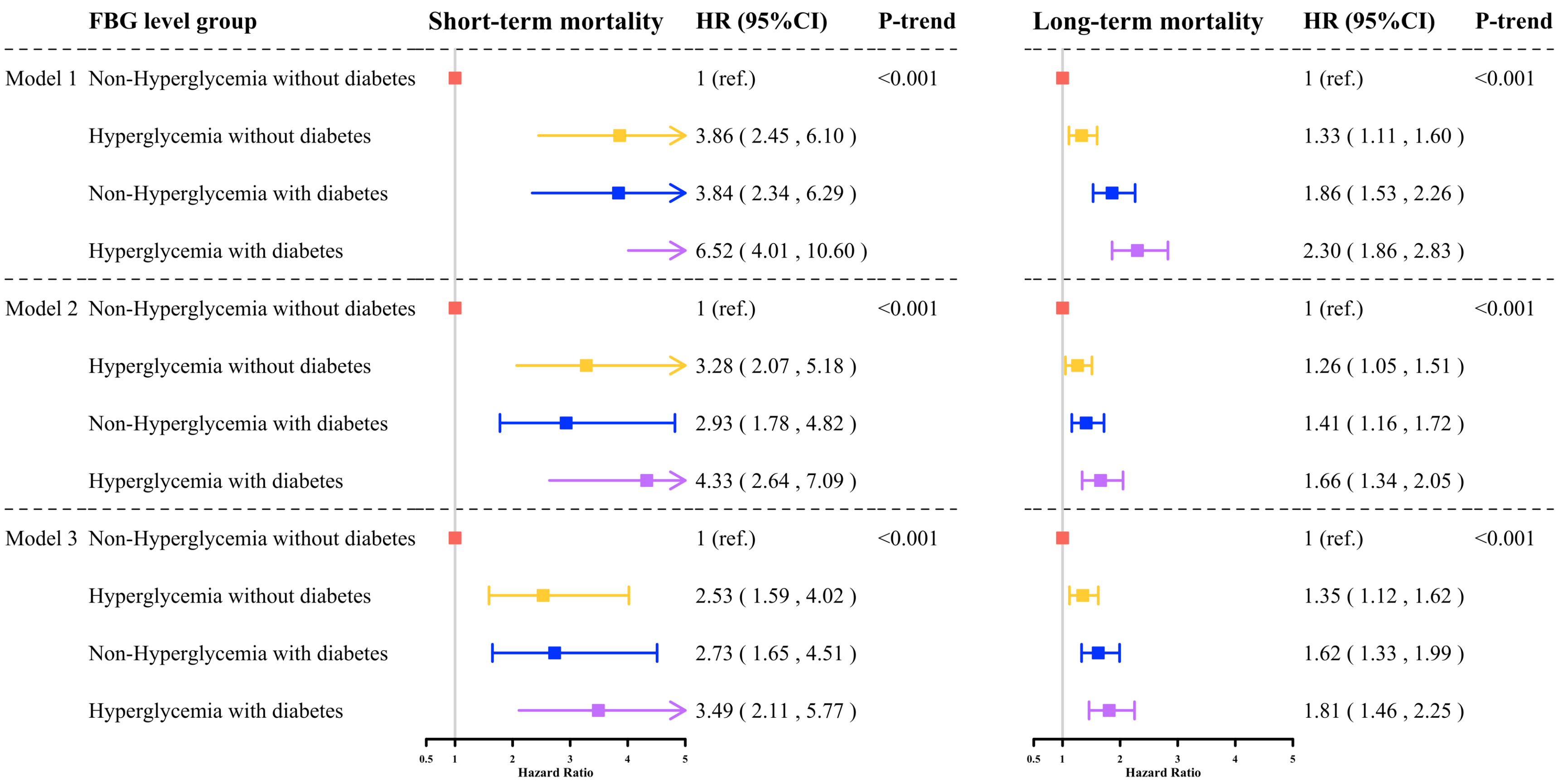

Model 1 included FBG categories only. Model 2 included FBG categories, age (≤ 55 and > 55 years), gender, ethnic, hypertension, diagnosis, Killip class (< II and ≥ II), and EF (< 40 and ≥ 40). Model 3 included FBG categories, age (≤ 55 and > 55 years), gender, Killip class (< II and ≥ II), ethnic, drinking, hypertension, COPD, liver disease, lung disease, diagnosis (NSTEMI and STEMI), EF (< 40 and ≥ 40), PCI, CABG, ACE inhibitor/ARB, and beta-blocker. CI = confidence interval; HR = hazard ratio.

Test for trend based on variable containing median value for each quintile.
